# No associations between C-reactive protein and spinal pain trajectories in children and adolescents (CHAMPS study-DK)

**DOI:** 10.1038/s41598-022-24587-7

**Published:** 2022-11-21

**Authors:** Amber M. Beynon, Niels Wedderkopp, Bruce F. Walker, Charlotte Leboeuf-Yde, Jan Hartvigsen, Bobby Jones, Ian Shrier, Chinchin Wang, Jeffrey J. Hébert

**Affiliations:** 1 Department of Chiropractic, Faculty of Medicine, Health and Human Sciences, Macquarie University, Sydney, NSW Australia; 2 College of Science, Health, Engineering and Education, Murdoch University, Perth, WA Australia; 3 Research Unit of Pediatrics, Department of Clinical Research, Faculty of Health Sciences, University of Southern Denmark, Odense, Denmark; 4 Faculty of Kinesiology, University of New Brunswick, Fredericton, New Brunswick, Canada; 5 Department for Regional Health Research, University of Southern Denmark, Odense, Denmark; 6 Center for Muscle and Joint Health, Department of Sports Science and Clinical Biomechanics, University of Southern Denmark, Odense, Denmark; 7 Chiropractic Knowledge Hub, Odense, Denmark; 8 Department of Psychiatry, University of Pittsburgh School of Medicine, Pittsburgh, PA USA; 9 Centre for Clinical Epidemiology, Lady Davis Institute, McGill University, Montreal, QC Canada; 10 Department of Epidemiology, Biostatistics and Occupational Health, McGill University, Montreal, QC Canada

**Keywords:** Pain, Biomarkers, Epidemiology

## Abstract

Preliminary evidence points to a link between C-reactive protein (CRP) and spinal pain in adults. However, there is a paucity of research in younger populations. Therefore, we aimed to determine associations between CRP and spinal pain in childhood and adolescence. We identified trajectories of spinal pain from childhood to adolescence and investigated the associations between CRP and trajectory subgroups. Six- to 11-year-old children from 13 primary schools, were followed from October 2008 and until 2014. High-sensitivity CRP collected at baseline (2008) was measured using serum samples. The outcome was the number of weeks with non-traumatic spinal pain between November 2008 and June 2014. We constructed a trajectory model to identify different spinal pain trajectory subgroups. The associations between CRP and spinal pain trajectory subgroups were modelled using mixed-effects multinominal logistic regression. Data from 1556 participants (52% female), with a mean age of 8.4 years at baseline, identified five spinal pain trajectory subgroups: “no pain” (55.3%), “rare” (23.7%), “rare, increasing” (13.6%), “moderate, increasing” (6.1%), and “early onset, decreasing” (1.3%). There were no differences in baseline high-sensitivity CRP levels between spinal pain trajectory subgroups. Thus, the heterogeneous courses of spinal pain experienced were not defined by differences in CRP at baseline.

## Introduction

Globally, spinal pain is the leading cause of disability^1^ and affects people across their life-course including children and adolescents^2,3^. Spinal pain is complex and has many possible contributors, including genetic, physical, and psychosocial factors^4^, and follows different trajectories in sufferers^5–7^. Low-grade persistent inflammation has been proposed as a biological mechanism for an array of health conditions^8,9^.

C-reactive protein (CRP) is a sensitive marker of inflammation in the human body. Adults generally have relatively stable levels of CRP with a median concentration of 0.8 mg/l, with occasional increased levels usually linked to infections or trauma^10^. CRP levels greater than 10 mg/L (clinical levels) are likely to indicate current infection and acute inflammation^11^. Sub-clinical levels of CRP, between 1–3 and 10 mg/L, have been associated with multiple factors for poor health^9^, such as metabolic syndromes^12,13^, coronary heart disease^14–17^, and diabetes^18,19^. In children, CRP has been correlated with cardiovascular risk factors such as fibrinogen, HDL-cholesterol, heart rate, and systolic blood pressure, as well as measurements of adiposity^20,21^.

There is also preliminary evidence that points to a potential link between CRP and spinal pain. For example, there is moderate quality evidence showing positive associations between CRP and the presence and severity of low back pain in adult populations^22,23^. Authors of a large cross-sectional population-based study (N = 15,322) reported that participants with obesity and high CRP levels had an almost three-fold increased odds of reporting low back pain than those without elevated CRP^24^. Data from cross-sectional studies in older or mixed-age participants from the general population indicate that higher levels of C-reactive protein may change the experience of spinal pain by altering underlying sensitisations^22,24–26^.

A 2020 systematic review investigating the relationship between systematic inflammation and neck pain suggested that future studies distinguish between traumatic and non-traumatic mechanisms of pain and analyse these groups separately^27^. From an aetiological perspective, it is logical to consider inflammation as a potential risk factor for non-traumatic spinal pain, and alternatively, as a potential mediator of the effect of trauma for traumatic spinal pain. This is because with increased inflammation there may be an inflammation-associated activation of the hypothalamic–pituitary–adrenal axis^28^. Dysregulation of this axis could lead to overactive responses to later psychosocial or mechanical stressors and overall hypersensitivity, resulting in an increased rate of reported pain^28^. Spinal pain is common in children and adolescents but is often nonspecific and benign^29,30^, with occurrences of traumatic spinal pain being rare^31^. Therefore, we focused on the potential relationship between an inflammatory marker such as CRP levels and non-traumatic spinal pain particularly in young populations.

In considering potential covariates, the literature also demonstrations a higher prevalence of back pain with female sex and advanced pubertal status^32,33^. There are mixed results regarding the relationship between body mass index (BMI) and spinal pain^32,33^. Physical activity has been found to be associated with future spinal pain^34^. Physical exercise has been shown to reduce CRP levels in adults, which could in turn lower the risk of coronary heart disease by moderating inflammation^35^. There are mixed results regarding sex differences, but females have been found to overall have higher levels of CRP in a general population^36,37^, additionally in children girls have also been found to have higher levels of CRP than boys^20^.

The aims of this study were to (1) define the non-traumatic spinal pain trajectories from the age of 6–17 years, and (2) investigate the associations between sub-clinical CRP levels at baseline and different courses of spinal pain from 6 to 17 years of age.

## Results

**Figure 1:**
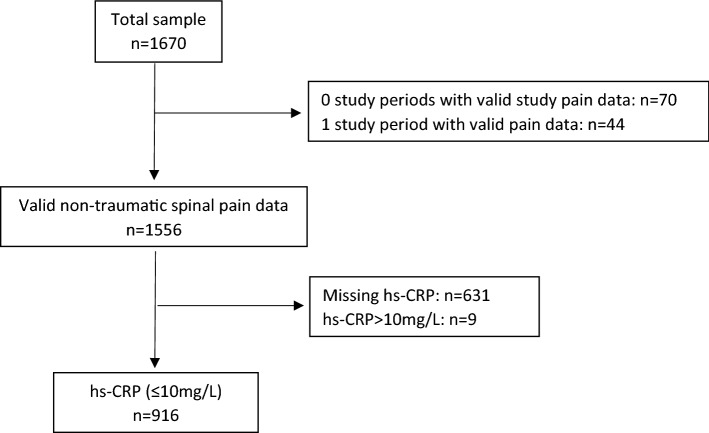
Participants flow diagram of participants with valid non-traumatic spinal pain and hs-CRP data from the CHAMPS Study-DK.

Overall, 1670 children were enrolled in the study. Data from 1556 participants were included in the non-traumatic spinal pain trajectory model, whereas data from 114 children were excluded as they had less than 2 study periods. At baseline, the study sample included 572 females (52%) and 527 males with a mean (SD) age of 8.4 (1.4) years. Nine hundred and sixteen participants met the inclusion criteria for hs-CRP (≤ 10 mg/L) (see Table 1 and Fig. 1). There was an extremely high response rate (96%) to the weekly spinal pain text messages^38^.

**Table 1:** Number, age, body mass index, and sex of participants at baseline, plus the number of participants meeting the hs-CRP inclusion criteria and participating in a study investigating the association between sub-clinical elevation of C-reactive protein and spinal pain trajectories in children and adolescents.

	All	Girls	Boys
Number of participants at baseline: n	1099	572	527
hs-CRP in ≤ 10 mg/L^a^, N (%)	916 (83.3%)	470 (82.2%)	446 (84.6%)
Age in years, mean (SD)	8.4 (1.4)	8.3 (1.4)	8.4 (1.4)
Body mass index in kg/m^2^, mean (SD)	16.4 (2.1)	16.4 (2.1)	16.3 (2.0)
MVPA in % of day, mean (SD)	8.1 (2.5)	7.4 (2.3)	9.0 (2.5)
hs-CRP in mg/L, mean (SD)	0.47 (0.86)	0.53 (0.88)	0.42 (0.83)

hs-CRP (≤ 10 mg/L)^a^: those with hs-CRP (≤ 10 mg /L), which indicates acute inflammation.

*SD* standard deviation, *MVPA* moderate-to-vigorous physical activity, *hs-CRP* high sensitivity C-reactive protein.

### Non-traumatic spinal pain trajectories

**Figure 2:**
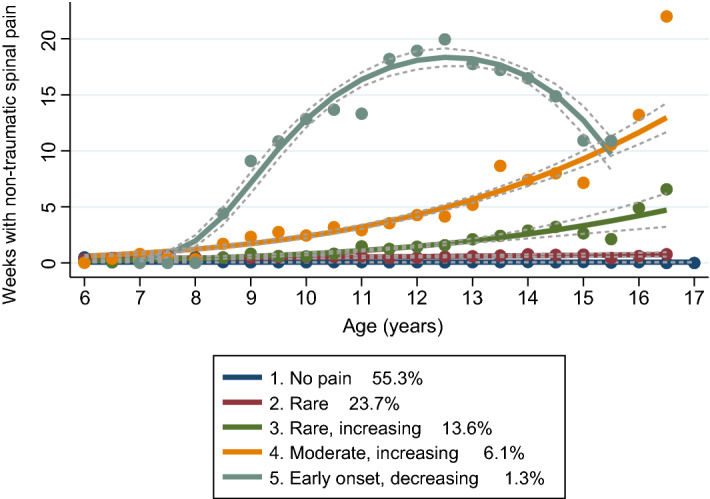
Non-traumatic spinal pain trajectories from 6 to 17 years of age and their prevalence (N = 1556). Points represent weeks with non-traumatic spinal pain in each 6-month period, dotted lines represent 95% confidence intervals.

The optimal fit was for five distinct non-traumatic spinal pain trajectory subgroups over the study period, which were labelled as follows (with the respective estimated group proportions): “no pain” (55.3%), “rare” (23.7%), “rare, increasing” (13.6%), “moderate, increasing” (5.9%), and “early onset, decreasing” (1.3%) (see Fig. 2). Our final models were consistent with all a priori diagnostic criteria: posterior probabilities were ≥ 80%, differences between the estimated group membership probability and the proportion of participants assigned to the group were small, and odds of correct classification exceeded 5.0 (see Supplementary Tables S1 and S2).

### Non-traumatic spinal pain trajectories and C-reactive protein levels

There were no differences in hs-CRP levels at baseline between the spinal pain trajectory subgroups (see Table 2). Female sex and level of health-related physical activity were found as significant covariates and included in the final regression model. Body mass index and pubertal status were not found to be significant covariates nor to make any changes to the magnitude of association, between spinal pain trajectories and hs-CRP, and were therefore not included in the final regression model.

**Table 2:** Mean hs-CRP estimates at baseline and risk ratios for each of the non-traumatic spinal pain trajectory subgroups compared to the “no pain” subgroup.

Spinal pain trajectory	Mean (SD)	RR (95% CI)
1 “No pain”	0.52 (0.98)	Reference
2 “Rare”	0.37 (0.43)	0.77 (0.57, 1.04)
3 “Rare, increasing”	0.47 (0.87)	1.00 (0.77, 1.31)
4 “Moderate, increasing”	0.44 (0.71)	0.93 (0.59, 1.48)
5 “Early onset, decreasing”	0.32 (0.33)	0.69 (0.19, 2.47)

Models adjusted for pubertal status, body mass index, health-related physical activity level (MVPA (moderate to vigorous physical activity)) and sex.

*hs-CRP* high sensitivity C-reactive protein, *SD* standard deviation, *RR* risk ratio, *95% CI* 95% confidence interval.

## Discussion

We identified five non-traumatic spinal pain trajectories in children from 6 to 17 years of age. Whilst there were some fluctuations in the amount of spinal pain within three of the trajectory groups ("rare, increasing” (13.6%), “moderate, increasing” (6.1%), and “early onset, decreasing” (1.3%)), three-quarters of children were members of the “no pain” (55.3%) or “rare pain” (23.7%) trajectory groups. These results are consistent with those of a recent systematic review, which found three common patterns of low back pain in adolescence and young adulthood also with the no pain category being most common^39^.

Contrary to our expectations, there were no significant differences in baseline hs-CRP levels between non-traumatic spinal pain trajectory subgroups. This finding agrees with a recent analysis of the Australian Raine Study, which also found no association between hs-CRP and low back pain with impact on daily living (including both traumatic and non-traumatic) in adolescence and young adulthood^7^. However, the current results conflict with a cross-sectional study of adolescents and adults (N = 15,322) that found elevated hs-CRP was associated with increased odds of self-reported (traumatic and non-traumatic) non-specific low back pain, particularly in individuals who were obese^24^. It may be that a relationship between CRP and spinal pain emerges only in older or obese populations and therefore not yet evident in the young. Further, this link could potentially relate to comorbidities in older age groups rather than just spinal pain and one inflammatory marker, CRP.

Female sex was found to be a significant covariate. Throughout the literature there has been an increased reported incidence and prevalence of back pain with female sex^32,33^, and girls have been found to have higher levels of CRP than boys^20^. It also appears the sex difference in the level of CRP accelerates during adolescence, which is potentially due to physiological processes that occurs during puberty^40^. However, within this current analysis pubertal status did not change the relationship between CRP and future spinal pain.

The main strengths of this study include its longitudinal design over 5.5 years with a large representative cohort of children, and the frequent and repeated measurements of non-traumatic spinal pain with high response rate. Spinal pain data were collected during weekly sampling windows, which likely minimised the potential for recall bias. The classification of spinal pain, based on clinical diagnoses, allowed us to isolate the pain attributable to non-traumatic aetiologies. There was a high rate (96%) of weekly spinal pain reporting^38^, and we applied random hotdeck multiple imputation methods to address missing outcome data^41^.

Limitations of our study could be that although our models considered several potential confounders, we did not apply formal graphical or counterfactual methods to examine different potential causal pathways between CRP and spinal pain. For example, we treated BMI as a confounder under the assumption that it is a common cause of both CRP level^20,42^ and spinal pain^32,33^. However, an alternative hypothesis is that BMI exists on the causal pathway between CRP and spinal pain and therefore acts as a mediator. Understanding these causal relations will require longitudinal data with repeated measures of each variable to identify any direct and indirect (e.g., BMI mediated) time-varying effects of CRP on spinal pain. Further, there could also be other potential confounders for the relationship between pain and CRP that were not considered within this analysis such as psychological factors (e.g., depression, anxiety, stress, early life adverse events) and socioeconomic factors. Although there was a high response rate to the weekly spinal pain reporting, approximately 40% of our sample did not have the exposure (CRP) measured at baseline, there may be a difference between the children who were included with CRP measurements meeting the inclusion criteria and the children who did not have the exposure (CRP) measurement. There are also limitations to our spinal pain outcome. We did not measure the severity or impact of the spinal pain, which represent pain-related outcomes that matter to patients. Future studies should consider applying causal inference methods if they have the required data available, and include a more diverse set of pain-related outcomes to further define if there are potential effects of CRP on spinal pain.

## Conclusion

We identified five non-traumatic spinal pain trajectories from 6 to 17 years of age. Whilst there were some fluctuations in the amount of spinal pain within three of the trajectory groups, the majority of children reported spinal pain rarely or not at all. We found no associations between hs-CRP and trajectories of non-traumatic spinal pain in children.

## Methods

### Study design and ethics permissions

We analysed data from the Childhood Health, Activity, and Motor Performance School Study Demark (CHAMPS study-DK)^43^. Six- to eleven-year-old pupils from 13 public primary schools were enrolled on a rolling basis from October 2008, and were followed until 2014^38,44^.

Ethics approval was obtained by the Regional Scientific Committee of Southern Denmark for the CHAMPS study-DK (ID S20080047) and the study was also registered with the Danish Data protection Agency, as stipulated by Danish law J.nr 2008-41-2240^38^. Written informed consent was obtained from every parent prior to commencement of the study. Every child and parent also gave verbal consent for all clinical examinations before the clinical examinations. All methods were performed in accordance with the relevant guidelines and performed in accordance with the Declaration of Helsinki. Ethics approval for the current analysis was also approved by Murdoch University Human Research Ethics Committee in Australia (Approval number: 2019/012).

### C-reactive protein

High-sensitivity CRP was measured using serum blood samples obtained at baseline (2008). Fasting blood samples were obtained in the morning (8.00–10.30 AM), stored on ice and transported within 4 h to a laboratory, where they were pipetted, centrifuged, and stored at – 80 °C^45^. High-sensitivity CRP (hs-CRP) refers to the lower detection limit of the assay compared to CRP. Participants with hs-CRP > 10 mg/L were excluded because this is likely to indicate current infection or acute inflammation rather than chronic inflammation^11^.

### Spinal pain outcome

Spinal pain was defined as “any report of pain in the neck, mid-back and/or lower back”. Spinal pain data were reported by parents each week over five and a half years (November 2008 to June 2014) via text messaging, except during the six-week summer holiday period and 1–2 week Christmas period^31^. If spinal pain was reported by text message, a clinician then followed up with the parent by a phone call within the week. If the pain was still present at the time of phone call, the child was then scheduled for physical examination within 1 week^31^. The physical examinations were performed by a medical doctor completing a residency as a paediatrician, and three physiotherapists with more than 10 years of experience in examining paediatric patients. If it was clinically indicated, children were then referred for further diagnostic investigation (e.g., diagnostic imaging, blood tests), orthopedic evaluation, or both^31^. If children obtained medical evaluation or treatment outside of the study (e.g., hospital), information about this was obtained through linked medical records^31^.

Children who were evaluated with physical examination (with or without diagnostic investigations) were diagnosed using International Classification of Diseases (ICD-10) coding (e.g., muscle strain, soft tissue pain, facet syndrome, torticollis)^46^. Diagnostic codes were classified as either traumatic or non-traumatic^31^. For the purpose of this study, we excluded all occurrences of diagnosed spinal pain arising from a traumatic aetiology (e.g., contusions, sprains, strains, fracture). Therefore, the spinal pain outcome comprised the total number of weeks of non-traumatic spinal pain. The follow-up data were grouped into 6-month periods starting from baseline. For each half year of follow-up, we used the total number of weeks with reported spinal pain to develop the trajectories over time. In order to contribute data to a study period, it was necessary for participants to be enrolled and responding to the text messages for a minimum of 60% of that period^31^.

### Eligibility criteria

Participants with hs-CRP > 10 mg/L were excluded because this is likely to indicate current infection or acute inflammation rather than chronic inflammation^11^. We also excluded three children with serious chronic health conditions that precluded their participation in the study activities; one child with dwarfism, one child with a congenital heart malformation, and one child with cerebral palsy. To be included in this current analysis participants needed to have two or more study periods with valid study pain data over the eleven study periods, with each study period it was necessary for participants to be enrolled and responding to the text messages for a minimum of 60% of that period.

### Covariates

Potential confounders or moderators included: sex, pubertal status, body mass index (BMI), and health-related physical activity (proportion of waking time in moderate-to-vigorous intensity physical activity [MVPA]). Demographic information was collected through a questionnaire at baseline. Puberty status was assessed through self-reported Tanner stages, as part of a structured interview. Tanner stages were reported on a scale of 1 to 5, with higher scores representing later pubertal status, based on self-assessments of pubic hair development in boys and breast development in girls^47,48^. Height was measured with a portable stadiometer (SECA 214, Seca Corporation, Hanover, MD, USA) to the nearest 0.5 cm, and body weight was measured using a calibrated Tanita BWB-800S digital scale (Tanita Corporation, Tokyo, Japan) to the nearest 100 g. Age- and sex-specific BMI categories for underweight, normal weight, overweight, and obese were calculated for all participants according to the International Obesity Task Force criteria^49^. Physical activity was measured objectively in September 2009 using Actigraph GTX3 accelerometers. Participants wore the accelerometer at the right hip, using a customised elastic belt, for seven consecutive days during waking hours (except when swimming or bathing). Data on physical activity were included if the participant accumulated at least ten hours of wear time on four or more days. We applied standard cut-points to identify moderate and vigorous physical activity intensities and isolated the proportion of the day in MVPA^43,50^. These covariates were chosen due to their associations with spinal pain^32–34^ and CRP^20,35–37^.

### Statistical analysis

Demographic data were reported descriptively including mean and standard deviation (SD) of hs-CRP. Missing data on non-traumatic spinal pain (for those who provided enough data to be analysed) were imputed by multiple imputation using random hot deck imputation^41^. Random hot deck imputation is a logic-based approach in which a pool of ‘donors’ with similar characteristics are identified and used to impute the missing value^51^. This method allows for the uncertainty of imputation to be accounted for. Five imputed datasets of spinal pain were created and used within the analyses to create the spinal pain trajectories, which has been reported in full previously^51^.

Trajectories of spinal pain frequency were generated using latent class growth analysis with spinal pain modelled as a function of age. Latent class growth analysis is a specialised application of finite mixture modelling that provides an empirical method of classifying meaningful subgroups of individuals, based on their patterns of change (i.e., trajectories) in outcome over time^52,53^. Contrary to the growth mixture modelling, this method uses maximum likelihood estimation to estimate and create an unknown distribution of trajectories across individuals^52^. In this way, models are well-suited to identify meaningful but previously unknown homogeneous subgroups (i.e., classes) that follow distinct trajectories within a heterogeneous population^11^.

We applied a zero-inflated Poisson distribution and applied equal weights across the five imputed datasets to generate a common model^31^. We included participants with two or more study periods with valid study pain data over the eleven study periods. For these analyses, single class models were constructed and the number of classes, and complexity of polynomial distributions (e.g., linear, quadratic, cubic) were increased until optimal models were identified^52^. A best model fit was selected using all available data estimating two to eight latent trajectory groups with zero-order, linear, quadratic, and cubic terms for each group. The initial modelling decisions were based on a combination of statistical and clinical judgments that were subsequently tested with several diagnostic approaches. We used the Bayesian Information Criterion (BIC) statistic best fit, then used clinical judgements to find clinically relevant trajectories. Models were then subsequently evaluated with a priori diagnostic criteria: (1) an average posterior probability of individual group membership of ≥ 70 per cent for each group; (2) close correspondence between the estimated probability of group membership and the proportion of participants assigned to each group based on the posterior probability; and (3) minimum odds of correct classification ≥ 5^52,53^.

We then investigated the association between baseline hs-CRP and membership in the different spinal pain trajectory subgroups by using multinominal logistic regression with robust standard errors. To account for the hierarchical nature of this school-based study, we included each child’s school class identifier as a random effect in all models. We reported the risk ratios (RR) with 95% confidence intervals. The “no spinal pain” trajectory was the reference category. Covariates were introduced into the model initially individually and then in combination, also assessing for any interaction effects between the variables. Covariates were included if they were statistically associated with spinal pain, or if they resulted in statistically significantly changes in the parameter estimate between hs-CRP and spinal pain. Data were analysed using Stata/SE version 15 (StataCorp. 2017. *Stata Statistical Software*: Release 15. College Station, TX: StataCorp LLC).

## Supplementary Information

Supplementary Information.

## Data Availability

Data are available from the CHAMPS Study Steering Committee upon reasonable request. Legal and ethical restrictions apply. Interested parties may contact Dr. Niels Christian Møller (nmoller@health.sdu.dk), and the following information will be required at the time of application: a description of how the data will be used, securely managed, and permanently deleted.
