# Supplementary material for: No associations between C-reactive protein and spinal pain trajectories in children and adolescents (CHAMPS study-DK)
Source: Sci Rep. 2022 Nov 21;12:20001. doi: 10.1038/s41598-022-24587-7 (PMC9678870; doi:10.1038/s41598-022-24587-7)
Supplement: Supplementary file 1 — Supplementary Information. [file 41598_2022_24587_MOESM1_ESM.docx]

**Supplementary Information: Model selection and Model fit diagnostic criteria for trajectories**

**Supplementary Table S1: Model selection based on Bayesian Information Criterion (BIC)**

| Number of groups | BIC^a^: Total number of observations | BIC^a^: Total number of participants |
| --- | --- | --- |
| 2 | -61843.81 | -61837.58 |
| 3 | -54867.53 | -54856.12 |
| 4 | -53700.79 | -53684.19 |
| 5 | **-52554.81** | **-52531.99** |
| 6 | -52228.38 | -52198.30 |
| 7 | -51905.85 | -51872.66 |
| 8 | -51789.11 | -51789.11 |
| ^a^BIC: Bayesian Information Criterion (large BIC indicates better fit) | | |

**Supplementary Table S2: Trajectory model diagnostics of Non-traumatic spinal pain trajectories**

| Trajectory group | Average posterior probability^a^: % | Odds of correct classification^b^ | Assigned membership: % | Estimated group proportions:  % (95% CI) |
| --- | --- | --- | --- | --- |
| 1 “No pain” | 86.8 | 5.3 | 61.6 | 55.3 (51.9, 58.6) |
| 2 “Rare” | 80.0 | 16.2 | 20.3 | 23.7 (20.1, 27.4) |
| 3 “Rare, increasing” | 85.6 | 50.3 | 10.9 | 13.6 (11.9, 15.3) |
| 4. “Moderate, increasing” | 90.4 | 154.1 | 6.0 | 6.1 (5.3, 6.9) |
| 5. Early onset, decreasing | 97.9 | 3667.1 | 1.3 | 1.3 (1.0, 1.5) |
| ^a^Lowest acceptable posterior probability 70%  ^b^Lowest acceptable odds of correct classification 5.0 | | | | |
